# [Retracted] Sirt1 inhibits HG-induced endothelial injury: Role of Mff-based mitochondrial fission and F-actin homeostasis-mediated cellular migration

**DOI:** 10.3892/ijmm.2024.5390

**Published:** 2024-06-25

**Authors:** Ruijie Qin, Lina Zhang, Dong Lin, Fei Xiao, Lixin Guo

Int J Mol Med 44: 89-102, 2019; DOI: 10.3892/ijmm.2019.4185

Following the publication of this paper, it was drawn to the Editor's attention by a concerned reader that certain of the immunochemistry data shown in Figs. 4K and 7G were strikingly similar to data appearing in different form in other research articles written by different authors at different research institutes that had either already been published, or were submitted for publication at around the same time.

Owing to the fact that contentious data in the above article had already been published elsewhere prior to its submission to *International Journal of Molecular Medicine,* the Editor has decided that this paper should be retracted from the Journal. The authors were asked for an explanation to account for these concerns, but the Editorial Office did not receive a satisfactory reply. The Editor apologizes to the readership for any inconvenience caused.

